# Anaphylaxis to a blood feeding leech

**DOI:** 10.1111/pai.70067

**Published:** 2025-03-22

**Authors:** Carmen H. Li, Maggie Jiang, Gabriele Gadermaier, Sebastian Kvist, Julia E. M. Upton, Xiaojun Yin, Jennifer A. Hoang, Mikhail Monteiro, Lisa Hung, Akash Kothari, Theo J. Moraes, Peter Vadas, Thomas Eiwegger

**Affiliations:** ^1^ Translational Medicine Program, Research Institute Hospital for Sick Children Toronto Ontario Canada; ^2^ Institute of Medical Science, Temerty Faculty of Medicine University of Toronto Toronto Ontario Canada; ^3^ Division of Clinical Immunology and Allergy St. Michael's Hospital Toronto Ontario Canada; ^4^ Department of Biosciences and Medical Biology Paris Lodron University Salzburg Salzburg Austria; ^5^ Department of Ecology and Evolutionary Biology University of Toronto Toronto Ontario Canada; ^6^ Department of Invertebrate Zoology Royal Ontario Museum Toronto Ontario Canada; ^7^ Swedish Museum of Natural History Stockholm Sweden; ^8^ Division of Immunology and Allergy, Food Allergy and Anaphylaxis Program The Hospital for Sick Children Toronto Ontario Canada; ^9^ Department of Pediatrics University of Toronto Toronto Ontario Canada; ^10^ Department of Immunology, Temerty Faculty of Medicine University of Toronto Toronto Ontario Canada; ^11^ Karl Landsteiner University of Health Sciences Krems Austria; ^12^ Department of Pediatric and Adolescent Medicine University Hospital St. Pölten St. Pölten Austria

**Abbreviations**
BAT: basophil activation test
BLAST: basic local alignment search tool
BSA: bovine serum albumin
Der f: *Dermatophagoides farinae*
Der p: *Dermatophagoides pteronyssinus*
EDTA: ethylenediaminetetraacetic acid
FcεRI: high‐affinity IgE receptor
FMLP: *N*‐formylmethionyl‐leucyl‐phenylalanine
GAPDH: glyceraldehyde 3‐phosphate dehydrogenase
HDM: house dust mite
IgE: immunoglobulin E
MD: *Macrobdella decora*
PBS: phosphate buffered saline
PMA: phorbol 12‐myristate 13‐acetate
PR: *Placobdella rugosa*
SPT: skin prick test
TBST: tris‐buffered saline with Tween

To the Editor,

About two‐thirds of known leech (phylum Annelida: subclass Hirudinea) species are parasitic, the remaining members being either predaceous or liquidosomatophagous.^1^ Anticoagulant salivary proteins from medicinal leeches have been implicated in anaphylaxis.^2^ However, leech allergen components are not well characterized.

We report a case of a 9‐year‐old boy at the time of presentation, with a history of atopic eczema, who was bitten by leeches while swimming in a freshwater lake at his family's cottage in Southern Ontario, Canada. Within minutes, he developed local swelling at the incision wound, followed by throat itching and upper lip blanching. He was immediately treated with oral diphenhydramine by the parents, but the reaction further progressed with generalized flushing, generalized urticaria, and dyspnea. He was taken to the emergency department, where he was treated with intramuscular epinephrine and intravenous steroids with resolution of clinical symptoms within 4 h. Aside from exertion through swimming and the cold temperature of the water, there were no other co‐factors. Prior to the incident, the boy had sustained several leech bites from the same lake with delayed localized swelling but no systemic reactions. In the absence of standardized extracts, prick‐to‐prick testing was completed with macerated frozen leeches (the glossiphoniid *Placobdella rugosa* ^3^ and the macrobdellid *Macrobdella decora* ^4^) collected from the same lake, which were positive. Within minutes of skin prick testing, the patient developed malaise and lightheadedness, requiring the administration of epinephrine with rapid resolution of symptoms. Two years later, he also developed urticaria, flushing, and throat discomfort to a hymenopteran sting, with skin testing positive to white faced hornet, paper wasp, yellow hornet, and yellow jacket. He also developed large local reactions to fire ants and black fly bites. Baseline serum tryptase was normal on both occasions (4.1 and 4.0 ng/mL). No acute serum tryptase was captured. Patient consent for publication was provided at St. Michael's Hospital (Toronto, Canada).

Skin prick tests (SPTs) to macerated frozen leeches were positive (≥3 mm diameter wheal) to both *P. rugosa* and *M. decora*. Intradermal skin tests to standardized hymenoptera venom were positive to venom from white‐faced hornet, paper wasp, yellow hornet, and yellow jacket.

To delineate the allergic sensitization spectrum of the patient, specific IgE was assessed via an allergen array (ALEX^2^) as described.^5^, ^6^ The patient had a positive sIgE (≥0.30 KU_A_/L) response to house dust mite (HDM) allergens Der f 1 (8.90), Der p 1 (10.71), Der p 23 (26.50), and paper wasp venom Polistes dominula (5.76), Pol d 5 (10.23), Vespula vulgaris (1.07), and Ves v 5 (6.69) (Table S1). Fire ant and honeybee venom were tested, but specific IgE to these venoms was undetectable. Specific IgE was reassessed at a follow‐up visit 3 years later, and the patient maintained a similar sensitization profile. Inhibition assays using the ALEX^2^ were performed with 1:4 (v/v) crude leech extracts from the two species, which led to a partial inhibition (~15%–60%) of IgE binding to HDM allergens and wasp venoms (Table S1).

Tissue samples from the anterior (containing salivary glands) and posterior parts of the leeches were prepared to distinguish reactivity to salivary proteins. Immunoblots displayed a positive IgE response to 140, 100, 70, and 37 kDa SDS gel bands, which were present in both species and in both anterior and posterior extracts (Figure 1A). Mass spectrometry analysis of IgE pull‐down and IgE binding protein bands found no substantial difference in proteins present in anterior or posterior extracts. Identified proteins from the leech were subjected to BLAST searches, and sequence identities to known allergens were determined (Table 1). Potential allergens included 140 kDa papain‐like proteins (UniProt accession T1EDM2) and 100 kDa paramyosin proteins (T1ECZ8 and T1FM89). T1EDM2 shares 21% sequence identity to Group 1 mite allergen Der p 1. T1ECZ8 and T1FM89 share approximately 35% sequence identity to Group 11 mite allergens Der p 11 and Der f 11. T1G8U8 is a 100 kDa calponin homolog with a 71% sequence identity with an allergenic alpha‐actinin from *Dermatophagoides farinae*.^7^ T1FNQ0 and T1EDJ2 are 70 kDa heat shock proteins that share 62%–76% sequence identity with hazelnut pollen Cor a 10 and mite allergens Der f 28 and Der p 28. Tropomyosin proteins are implicated as major allergens in crustaceans and as minor allergens in mites and cockroaches.^8^ T1FZT0 is a 37 kDa tropomyosin with a 56% sequence identity to Der p 10. T1FMP1 is a 37 kDa fructose‐bisphosphate aldolase with 88% sequence identity with an allergen identified in the saliva of the biting midge *Forcipomyia taiwana*.^9^ The full list of identified proteins is in Table S2.

**FIGURE 1:**
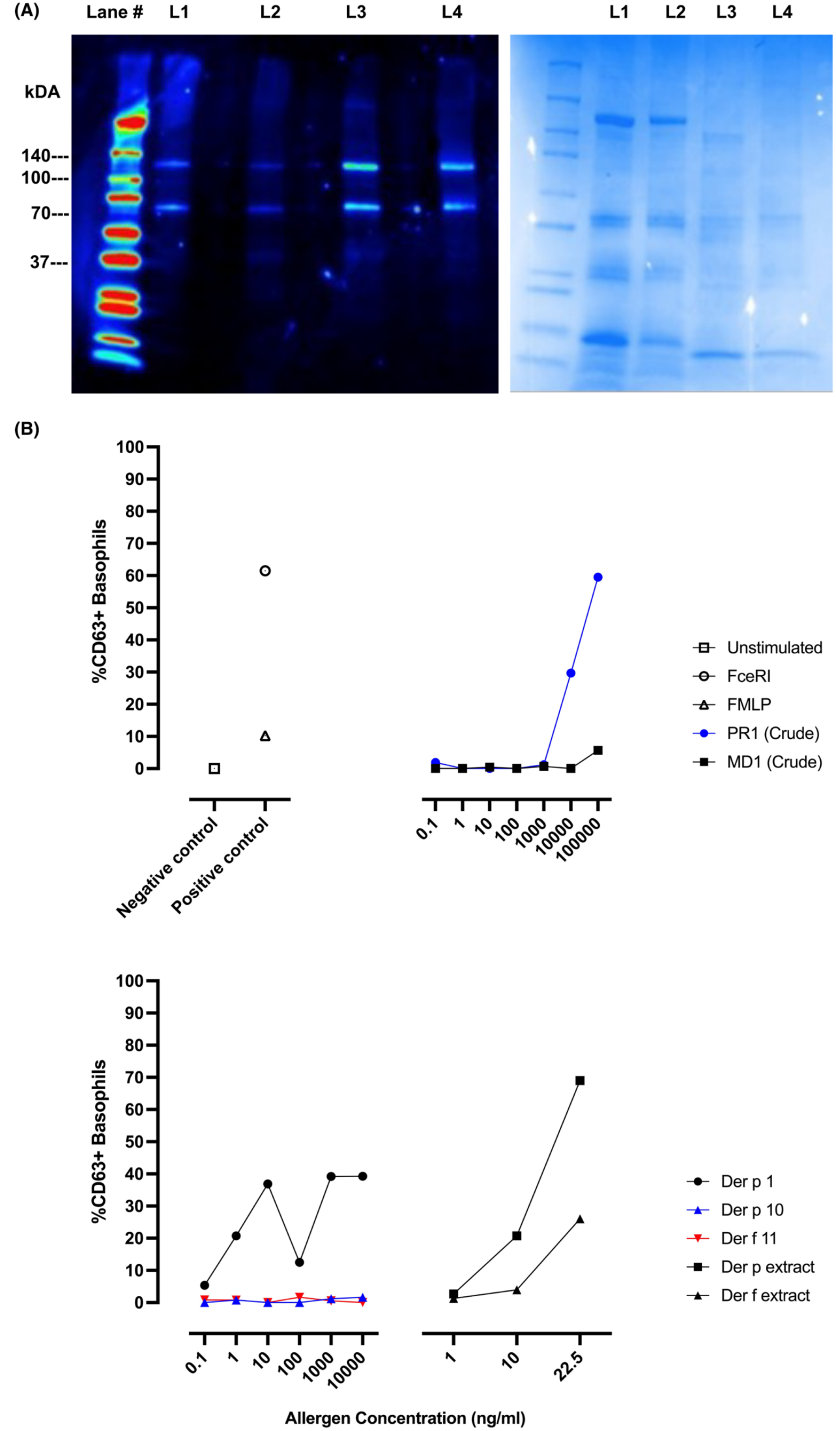
IgE‐mediated response to leech and house dust mite. (A) 20 μg protein/lane SDS‐PAGE IgE immunoblot and Coomassie. Lane 1: *Macrobdella decora* (MD) anterior, L2: MD posterior, L3: *Placobdella rugosa* (PR) anterior, L4: PR posterior. (B) Basophil activation to PR (Crude), MD (Crude), Der p 1, Der p 10, Der f 11, *Dermatophagoides pteronyssinus* extract, and *Dermatophagoides farinae* extract. FMLP, *N*‐formylmethionine‐leucyl‐phenylalanine.

**TABLE 1:** Potential leech reactive proteins identified by mass spectrometry of IgE‐reactive protein bands identified by immunoblot.

Leech proteins	Protein identity	Allergen protein family	Homologous allergens/proteins
140 kDa, papain‐like, transglutaminase‐like uncharacterized protein	T1EDM2	Group 1 mite allergens, papain	Der p 1, Der f 1
100 kDa, Myosin_tail_1 domain‐containing protein	T1ECZ8, T1FM89	Group 11 mite allergens, paramyosin	Der p 11, Der f 11
100 kDa, actin binding, calponin‐homology, EF‐hand, coiled coil	T1G8U8	Alpha‐actinin	Drosophila alpha‐actinin^7^
70 kDa, heat‐shock protein	T1FNQ0, T1EDJ2	Hsp70 proteins	Cor a 10, Der p 28, Der f 28
37 kDa, glyceraldehyde‐3‐phosphate dehydrogenase	T1FMX4	Glyceraldehyde 3‐phosphate dehydrogenase (GAPDH)	Chicken GAPDH
37 kDa, tropomyosin	T1FZT0	Tropomyosin	Der p 10, Der f 10
37 kDa, fructose biphosphate aldolase	T1FMP1	Fructose‐bisphosphate aldolase	*Forcipomyia taiwana* ^9^

IgE‐mediated response to leech extract was confirmed using the basophil activation test (BAT). BAT confirmed reactivity (≳5% CD63^+^ basophils) to pooled anterior and posterior crude leech, HDM, and Der p 1 (Figure 1B).

Exposure to leech bites is common in North America. Leech allergen components have not been well characterized and there is a conspicuous lack of data regarding the presence of hirudin‐like proteins in the saliva of North American leeches, such that the major allergens in North American leeches may well be distinct from those of their European counterparts.

In our clinical case, the patient developed allergic symptoms following a leech bite and had positive SPT and BAT responses to crude leech extract, which confirm the allergic phenotype. The IgE assays and immunoblots indicated that the proteins to which the patient was sensitized were not specific to leech salivary proteins and were present in both the anterior and posterior tissue of the two leech species. We describe leech allergen candidates distinct from previously attributed leech salivary proteins that share low to high sequence identity (21%–88%) with allergens from HDM, invertebrate allergens, and pollen.

Partial cross‐reactivity between leech, HDM, and wasp venoms was demonstrated using inhibition assays. Co‐sensitization to Hymenoptera wasp venom has been observed in patients with anaphylaxis to leech in literature.^10^ Cross‐reactivity may contribute to this observation, though more studies are needed to explore this relationship and clarify which allergenic source was the primary sensitizer. In conclusion, this report describes a novel trigger of systemic allergic reactions due to leech exposure, not related to leech salivary or anticoagulant proteins.

## AUTHOR CONTRIBUTIONS

**Carmen H. Li:** Investigation; methodology; writing – original draft; data curation; conceptualization; formal analysis; visualization; writing – review and editing; validation. **Maggie Jiang:** Investigation; methodology; writing – review and editing; writing – original draft. **Gabriele Gadermaier:** Writing – review and editing; software; methodology; formal analysis; data curation; validation; investigation; visualization. **Sebastian Kvist:** Writing – review and editing; methodology; investigation; data curation; validation; visualization. **Julia E. M. Upton:** Writing – review and editing; validation. **Xiaojun Yin:** Writing – review and editing; methodology; investigation; data curation; visualization. **Jennifer A. Hoang:** Writing – review and editing; methodology; investigation; data curation. **Mikhail Monteiro:** Writing – review and editing; methodology; investigation; data curation. **Lisa Hung:** Writing – review and editing; methodology. **Akash Kothari:** Writing – review and editing; methodology. **Theo J. Moraes:** Writing – review and editing. **Peter Vadas:** Supervision; resources; data curation; writing – review and editing; conceptualization; funding acquisition; investigation; methodology; validation; writing – original draft. **Thomas Eiwegger:** Supervision; resources; data curation; project administration; writing – review and editing; methodology; conceptualization; funding acquisition; investigation; writing – original draft; validation.

## FUNDING INFORMATION

This work was supported by the Hospital for Sick Children (SickKids Food Allergy and Anaphylaxis Program, start‐up funds by the SickKids Research Institute and Department of Pediatrics, Restracomp Graduate Scholarship to CHL, AK, and LH), and St. Michael's Hospital Division of Clinical Immunology and Allergy. CHL, AK, and LH are recipients of the Canadian Institutes of Health Research (CIHR) Frederick Banting and Charles Best Canada Graduate Scholarship.

## CONFLICT OF INTEREST STATEMENT

TE reports to act as local PI for company‐sponsored trials by DBV Therapeutics, Greer Stallergens, and sub‐investigator for Regeneron and ALK‐Abelló. He is Co‐Investigator or scientific lead in three investigator‐initiated oral immunotherapy trials supported by the SickKids Food Allergy and Anaphylaxis Program and serves as an associate editor for Allergy. He/his laboratory received unconditional/in‐kind contributions from Macro Array Diagnostics and an unrestricted grant from ALK‐Abelló. He holds advisory board roles for ALK‐Abelló, VAMED, Nutricia/Danone, and Aimmune. TE reports lecture fees from Novartis, Thermo Fisher, Nutricia/Danone, Aimmune, and ALK‐Abelló. PV serves as PI for trials of Avapritinib (Blueprint Medicines) and of CGT9486 (Cogent Biosciences) for systemic mastocytosis. He is a speaker and holds advisory board roles for Aralez, Miravo, Pediapharm, BioCryst, CSL Behring, and Sanofi. JEMU reports grants and personal fees from ALK‐Abelló, personal fees from Bausch Health, Pfizer, Pharming Group N.V, grants from DBV Technologies, grants from Food Allergy Anaphylaxis Programme (SickKids), CIHR, Board of Directors at Canadian Society of Allergy and Clinical Immunology; Healthcare Advisory Board Food Allergy Canada. GG reports advisory board fees from HESI and expert fees from EFSA, non‐related to this work. All other authors report no conflict of interest.

## Supporting information


Appendix S1
