# Supplementary material for: Anaphylaxis to a blood feeding leech
Source: Pediatr Allergy Immunol. 2025 Mar 22;36(3):e70067. doi: 10.1111/pai.70067 (PMC11928907; doi:10.1111/pai.70067)
Supplement: Supplementary file 1 — Appendix S1 [file PAI-36-e70067-s001.docx]

**Supplementary:**  Anaphylaxis to a blood feeding leech

Authors: Carmen H. Li MSc^1,2^*, Maggie Jiang MD^3^*, Gabriele Gadermaier PhD^4^, Sebastian Kvist​ PhD^5,6,7^ Julia E. M. Upton MD^8,9^, Xiaojun Yin PhD^1^, Jennifer A. Hoang MSc MDS^1,10^, Mikhail Monteiro BSc^1^, Lisa Hung PhD^1,10^, Akash Kothari MSc^1,2^, Theo J. Moraes MD PhD^1,9^, Peter Vadas MD PhD^3,10^**, Thomas Eiwegger MD PhD^1,10,11,12^**

*Co-first author

**Co-senior author

(1) Translational Medicine Program, Research Institute, Hospital for Sick Children, Toronto, ON, Canada

(2) Institute of Medical Science, Temerty Faculty of Medicine, University of Toronto, Toronto, ON, Canada

(3) Division of Clinical Immunology and Allergy, St. Michael’s Hospital

(4) Department of Biosciences and Medical Biology, Paris Lodron University Salzburg, Salzburg, Austria

(5) Department of Ecology and Evolutionary Biology, University of Toronto, ON, Canada

(6) Department of Invertebrate Zoology, Royal Ontario Museum, Toronto, ON, Canada

(7) Swedish Museum of Natural History, Stockholm, Sweden

(8) Division of Immunology and Allergy, Food Allergy and Anaphylaxis Program, The Hospital for Sick Children

(9) Department of Pediatrics, University of Toronto, Toronto, ON, Canada

(10) Department of Immunology, Temerty Faculty of Medicine, University of Toronto, Ontario, Toronto, Canada

(11) Karl Landsteiner University of Health Sciences, Krems, Austria

(12) Department of Pediatric and Adolescent Medicine, University Hospital St. Pölten, St. Pölten, Austria

**Methods:**

**Skin Prick Testing (SPT):**

Leeches (identified as *Placobdella rugosa* and *Macrobdella decora*) were collected and frozen from the freshwater lake where the patient was bitten. The leeches were then mashed, mixed with sterile saline, and used for skin prick testing (SPT) by prick-to-prick method with steep lancets at St Michael’s Hospital. Saline was used as the negative control, and histamine was used as the positive control.

The patient’s community allergist also performed intradermal skin testing on standardized wasp, honeybee, yellow jacket, yellow hornet, and white-faced hornet venom manufactured by Omega Laboratories (Cleveland, Ohio, United States).

**Sample collection and storage:**

Patient whole blood and serum were collected using ethylenediaminetetraacetic acid (EDTA) blood tubes in 2021 following anaphylaxis to leech. A second sample was collected in 2024 at a follow-up clinic visit. Serum was stored at –80ºC.

Dr. Sebastian Kvist, a local invertebrate and leech expert, assessed the lake in which the patient had experienced an allergic reaction to leech species. Then, live leeches (*Placobdella rugosa*, *Macrobdella decora)* were collected at the cottage lake where the patient’s leech bite occurred. The anterior and posterior sections of each leech were excised. The anterior sections contained the salivary glands and muscle tissue. The posterior section only contained muscle tissue. Crude leech protein extracts were individually prepared with each anterior and posterior section (T-PER™ Tissue Protein Extraction Reagent, Thermo Fisher) with protease inhibitors (Halt™ Protease Inhibitor Cocktail, Thermo Fisher) and stored at –80ºC.

**Molecular Diagnosis of IgE:**

Allergen-specific IgE was measured with the Allergy Xplorer (ALEX^2^) array kit, as described in the main text. Positive sensitizations were defined as a specific IgE of ≥0.30 kU_A_/L.

**IgE inhibition:**

Placobdella rugosa and Macrobdella decora crude extract were incubated with patient serum in a 1:4 (v/v) dilution for one hour before following the ALEX2 manufacturer protocol.

**Protein extraction and identification:**

*SDS-PAGE and Immunoblot:*

Protein concentration of crude extracts was determined using Pierce™ Rapid Gold BCA Protein Assay Kit (Thermo Fisher). The SDS-PAGE was completed using 4–20% Mini-PROTEAN® TGX™ Precast Protein Gels (BioRad) and Precision Plus Protein™ WesternC™ Blotting Standards (BioRad) on the Mini Protean Tetra Gel 2 Gel System (BioRad). 20 ug of the anterior and posterior extracts of the two species were run independently on one set of gels. The gels were Coomassie stained (0.1% Coomassie R-250 in 40% methanol 10% acetic acid) and imaged using BioRad ChemiDoc MP. Gels were transferred to Immuno-Blot LF PVDF membrane (BioRad) using the Mini Trans Blot Cell Module (BioRad).

The membrane was blocked with 5% Bovine serum albumin/Tris Buffered Saline with Tween (BSA/TBST) buffer for one hour. The blot was then incubated overnight at 4ºC with 1:50 (v/v) patient serum and 5% BSA/TBST. After washing, the blot was incubated with Invitrogen 1 mg Goat Anti-human IgE antibody at 1:20000 (v/v) in 5% BSA/TBST for one hour at room temperature. SuperSignal™ West Pico PLUS Chemiluminescent Substrate (Thermo Scientific) was prepared according to manufacturer's instructions before being added to the blot for image capture with the LI-COR Odyssey Imager. Cut gel bands corresponding to IgE reactive proteins were submitted for mass spectrometry.

*IgE Pull-Down Assay:*

50 μl Dynabeads M-280 sheep anti-mouse IgG (Thermo Fisher) were coupled with 4 ug of Mouse monoclonal anti-IgE antibody (Cat no. ab7295, Abcam) for 1 hour at room temperature. The conjugated beads were then washed and incubated with patient serum 1:5 v/v in phosphate buffered saline (PBS) overnight at 4 °C. Conjugated beads were then washed and incubated with 20 μg crude extract for three hours at room temperature. Beads were washed and submitted for mass spectrometry.

**Mass spectrometry:**

In-gel cut bands and IgE pulldown assay beads were submitted to the SPARC BioCentre for Molecular Analysis (SickKids) facility for protein identification (Thermo Scientific Exploris 480). Samples were reduced using 10 mM DTT (60°C, 1 hour) followed by alkylation (55 mM iodoacetamide, at room temperature for 20 minutes in the dark). Samples were digested using trypsin (Pierce, ~ 600 ng per sample, 37°C, overnight). Proteins were extracted (5% Formic acid/ 100% Acetonitrile) and submitted for analysis. Proteome Discoverer version: 2.5.0.400 software was used for protein identification and quantification. Proteins were searched against the genome uniprot-3A6412_Helobdella_robusta_CalifornianLeech_03022022.fasta.

**Determination of protein homologies:**

For mass spectrometry-based analysis of the cut bands, proteins with ≥4 identified peptides were considered. The highest quantitative values (normalized total protein) and appropriate mass range, in combination with detection in the IgE pulldown assay, were used as parameters for identifying potential leech allergens. Identified proteins were submitted to a BLAST search. Sequence identities to known allergens and related proteins were analyzed.

**Basophil activation testing:**

Basophil activation test (BAT) was performed according to the manufacturer protocol (FlowCAST BÜHLMANN, Switzerland). Whole blood was stimulated with extracts or allergens for 15 minutes and CD63 expression on basophils was measured. Basophils were gated SSC^low^ and CCR3^+^. Activated basophils were gated CD63^+^. Extracts included: crude leech extracts (*Placobdella rugosa,* *Macrobdella decora), Dermatophagoides pteronyssinus* (Buhlmann), *Dermatophagoides farinae* (Buhlmann), Der p 1 (Cat no. NA-DP1-1, InBio), Der p 10 (Cat no. RE-DP10-1, InBio), Der f 11 (Cat no. MBS1436079, BioSource), Rabbit aldolase (Sigma Aldrich). Allergen specific basophil activation was assessed along a log scale of allergen concentrations (0.01-1000 ng/ml, **Figure 1B**). Flow cytometric analysis was performed with CytoFLEX (Beckman Coulter, United States).

**Table S1**. IgE Inhibition Assay

| **Baseline Sensitization** | | | | **IgE Inhibition with Crude Leech Extract Relative to 2024 Sensitization** | | | |
| --- | --- | --- | --- | --- | --- | --- | --- |
| **Source** | **Extract/**  **Component** | **2021**  **kU_A_/L** | **2024**  **kU_A_/L** | **Source** | **Extract/**  **Component** | **MD 1:4** | **PR 1:4** |
| American House Dust Mite | Der f 1 | 8.9 | 3.70 | American House Dust Mite | Der f 1 | 1.74  (-52.9%) | 2.41  (-34.8%) |
| European House Dust Mite | Der p 1 | 10.71 | 8.05 | European House Dust Mite | Der p 1 | 6.70  (-16.7%) | 6.91  (-14.1%) |
| European House Dust Mite | Der p 23 | 26.50 | 18.34 | European House Dust Mite | Der p 23 | 12.58  (-31.4%) | 13.93  (-24.0%) |
| Hornet | Dol spp | ≤0.1 | 0.33 | Hornet | Dol spp | 0.21  (-36.3%) | 0.12  (-63.6%) |
| Paper wasp venom | Pol d | 5.76 | 2.09 | Paper wasp venom | Pol d | 1.34  (-35.8%) | 1.75  (-16.2%) |
| Paper wasp venom | Pol d 5 | 10.23 | 6.45 | Paper wasp venom | Pol d 5 | 4.83  (-25.1%) | 5.47  (-15.1%) |
| Wasp Venom | Ves v | 1.07 | 0.24 | Wasp Venom | Ves v | 0.18  (-25.0%) | 0.12  (-50.0%) |
| Wasp Venom | Ves v 5 | 6.69 | 2.29 | Wasp Venom | Ves v 5 | 1.49  (-34.9%) | 2.01  (-12.2%) |

**Table S2.** Leech Proteins Identified by Mass Spectrometry

| **Uniprot Ascension Number** | **Description** | **Allergen Family** | **Notes from AllFam Allergen Database** |
| --- | --- | --- | --- |
| T1EDM2 | 140 kDa Papain-like, Transglutaminase-like  Uncharacterized protein OS=Helobdella robusta OX=6412 GN=20194674 PE=4 SV=1 | Papain | Members of the papain family are wide-spread in nature, having been found in baculovirus, bacteria, yeast, plants and animals. Despite structural similarities regarding the residues surrounding the catalytic site, the cysteine proteases have only low levels of overall sequence similarity. Papain-like cysteine proteinases are synthesized as inactive proenzymes with N-terminal propeptide regions. The propeptide plays important roles as inhibitor of enzymatic activity and for the correct folding of the newly synthesized protein. The mature enzymes are generally 25-28 kDa in size.  A number of allergens have been identified belonging to the papain family including group 1 mite allergens such as the major dust mite allergen Der p 1 and food allergens such as actinidin, the major allergen from kiwifruit, bromelain from pineapple, ficin from fig and papain from papaya. Gly m Bk30K (P34), a major allergen from soybean seed storage vacuoles, shows sequence similarity to papain-like proteases but lacks enzymatic activity. |
| T1ECZ8 | 100 kDa Myosin_tail_1 domain-containing protein OS=Helobdella robusta OX=6412 GN=20194450 PE=4 SV=1 | Myosin | Myosin is a multi-subunit complex made up of two heavy chains (around 200 kDa) and four light chains (around 20 kDa). It is a fundamental contractile protein found in all eukaryotic cell types. Most myosin molecules are composed of a head, neck, and tail domain. The head domain binds the actin filament and uses ATP hydrolysis to generate force and to "walk" along the filament. The neck domain acts as a linker and as a lever arm for transducing force generated by the catalytic motor domain. It also serves as a binding site for myosin light chains, which are members of the (EF hand family). The tail domain forms a coiled coil and mediates interaction with cargo molecules and other myosin subunits. The coiled-coil is composed of the tails from two molecules of myosin and assembles into the thick filament in muscle cells.  Group 11 mite allergens, also termed paramyosin (Blo t 11, Der f 11, Der p 11, Tyr p 11), size around 100 kDa |
| T1FM89 | 100 kDa Myosin_tail_1 domain-containing protein OS=Helobdella robusta OX=6412 GN=20209938 PE=4 SV=1 | Myosin |  |
| T1G8U8 | 100 kDa Actin binding, Calponin-homology, EF-hand, coiled coil  Uncharacterized protein OS=Helobdella robusta OX=6412 GN=20217495 PE=3 SV=1 |  | Relevance to allergy unclear. |
| T1FNA0 | 70 kDa Intermediate filament rod/LTD/coiled coil  Uncharacterized protein OS=Helobdella robusta OX=6412 PE=3 SV=1 |  | Involvement of this protein family in allergy unclear. |
| T1FML6 | 70 kDa Intermediate filament rod/LTD/coiled coil  Uncharacterized protein OS=Helobdella robusta OX=6412 GN=20210065 PE=3 SV=1 |  |  |
| T1ELF0 | 70 kDa Intermediate filament rod/LTD/coiled coil  Uncharacterized protein OS=Helobdella robusta OX=6412 GN=20197400 PE=3 SV=1 |  |  |
| T1FNQ0 | 70 kDa heat-shock protein 70  Uncharacterized protein OS=Helobdella robusta OX=6412 GN=20210447 PE=3 SV=1 | Hsp70 | Hsp70 proteins are ubiquitous chaperones that catalyze protein folding driven by hydrolysis of ATP.  Allergens belonging to the Hsp70 family are found in a heterogeneous range of sources. Several fungal allergens belong to this family such as Mala s 10 from *Malassezia sympodialis* and Alt a 3 from *Alternaria alternata*. In addition, the hazel pollen allergen Cor a 10 and Der f 28 from the mite *Dermatophagoides farinae* belong to this family. |
| T1EDJ2 | 70 kDa heat-shock protein 70  Uncharacterized protein OS=Helobdella robusta OX=6412 GN=20194644 PE=3 SV=1 | Hsp70 |  |
| T1FME7 | 37 kDa Actin  Uncharacterized protein OS=Helobdella robusta OX=6412 GN=20197421 PE=3 SV=1 | Actin | Actin is reported as allergen in carpet clam. |
| T1G9A8 | 37 kDa Actin  Uncharacterized protein OS=Helobdella robusta OX=6412 GN=20217655 PE=3 SV=1 | Actin |  |
| T1FNP2 | 37 kDa uncharacterized protein, homology with bacteria  Uncharacterized protein OS=Helobdella robusta OX=6412 GN=20210439 PE=4 SV=1 |  | Involvement in allergy unclear. |
| T1FMX4 | 37 kDa Glyceraldehyde-3-phosphate dehydrogenase OS=Helobdella robusta OX=6412 GN=20210173 PE=3 SV=1 | Glyceraldehyde 3-phosphate dehydrogenase | Glyceraldehyde 3-phosphate dehydrogenase (GAPDH) is an enzyme of about 37 kDa that catalyzes the sixth step of glycolysis, the conversion of glyceraldehyde 3-phosphate to 1,3-bisphosphoglycerate. In addition to this long-established metabolic function, GAPDH has recently been implicated in several non-metabolic processes, including transcription activation, initiation of apoptosis, and ER to Golgi vesicle shuttling.  Tri a 34, the GAPDH from wheat, was identified as a minor allergen for patients with baker's asthma.  This allergen family is frequently identified when performing MS analysis with allergenic extract, limited relevance for allergy. |
| T1FZT0 | 37 kDa Tropomyosin  Uncharacterized protein OS=Helobdella robusta OX=6412 GN=20214328 PE=3 SV=1 | Tropomyosin | Tropomyosins are present in muscle and non-muscle cells. In striated muscle, they mediate the interactions between the troponin complex and actin so as to regulate muscle contraction. The role of tropomyosin in smooth muscle and non-muscle tissues is not clear. Tropomyosin is an α-helical protein that forms a coiled-coil structure of two parallel helices containing two sets of seven alternating actin binding sites.  Tropomyosins were identified as minor inhalative allergens in arthropods (mites, cockroaches) and as major food allergens in crustaceans and mollusks. Tropomyosin is also a minor allergen in the fish parasite *Anisakis simplex*. Vertebrate tropomyosins seem to be non-allergenic. Due to their repetitive coiled-coil structures, tropomyosins retain their IgE binding ability even after prolonged heating or partial digestion. Tropomyosin sequences are highly conserved, which explains the frequent cross-sensitization among distantly related allergen sources. |
| T1FMP1 | 37 kDa Fructose-bisphosphate aldolase OS=Helobdella robusta OX=6412 GN=20210090 PE=3 SV=1 | Fructose-bisphosphate aldolase | Fructose-bisphosphate aldolase, often just aldolase, is an enzyme catalyzing a reversible reaction that splits the aldol, fructose 1,6-bisphosphate, into the triose phosphates dihydroxyacetone phosphate and glyceraldehyde 3-phosphate. Aldolase can also produce DHAP from other (3S,4R)-ketose 1-phosphates such as fructose 1-phosphate and sedoheptulose 1,7-bisphosphate. Gluconeogenesis and the Calvin cycle use the reverse reaction. Aldolases are divided into two classes by mechanism. Members of both families fold into a TIM barrel but show little sequence similarity. With few exceptions, only class I proteins have been found in animals, plants, and green algae while only class II proteins have been found in fungi. Both classes have been found widely in other eukaryotes and in bacteria.  Aldolases were identified as major fish allergens with limited cross-reactivity. |
| T1FN30 | 25 kDa EF-hand, calcium binding  Uncharacterized protein OS=Helobdella robusta OX=6412 GN=20210229 PE=4 SV=1 |  | Myosin regulating proteins. |
| T1G2B6 | 15 kDa EF-hand domain-containing protein OS=Helobdella robusta OX=6412 GN=20215214 PE=4 SV=1 |  |  |
